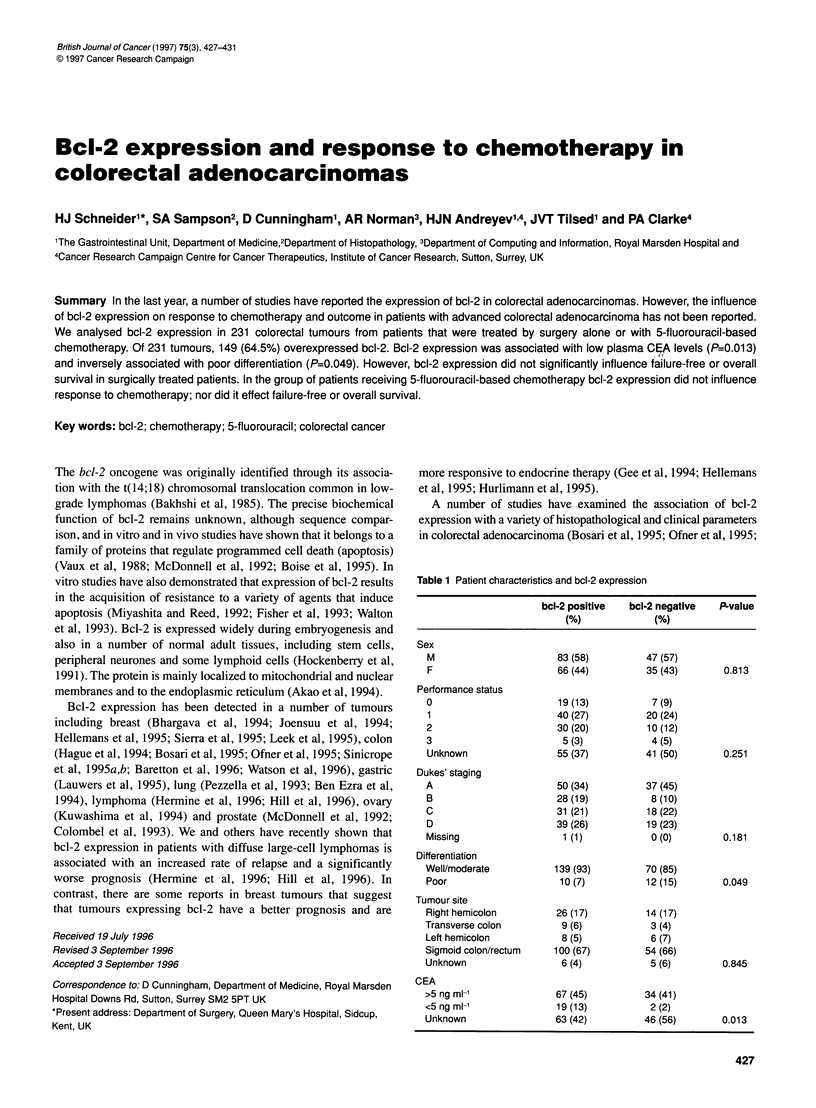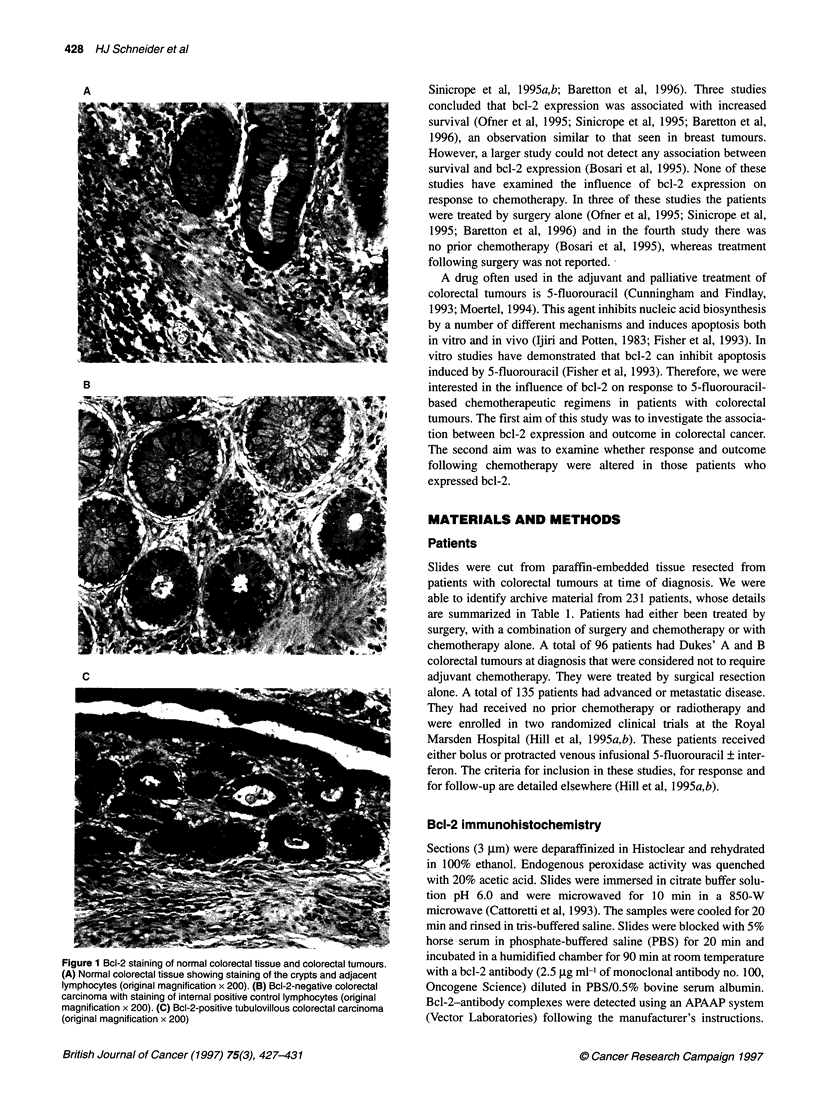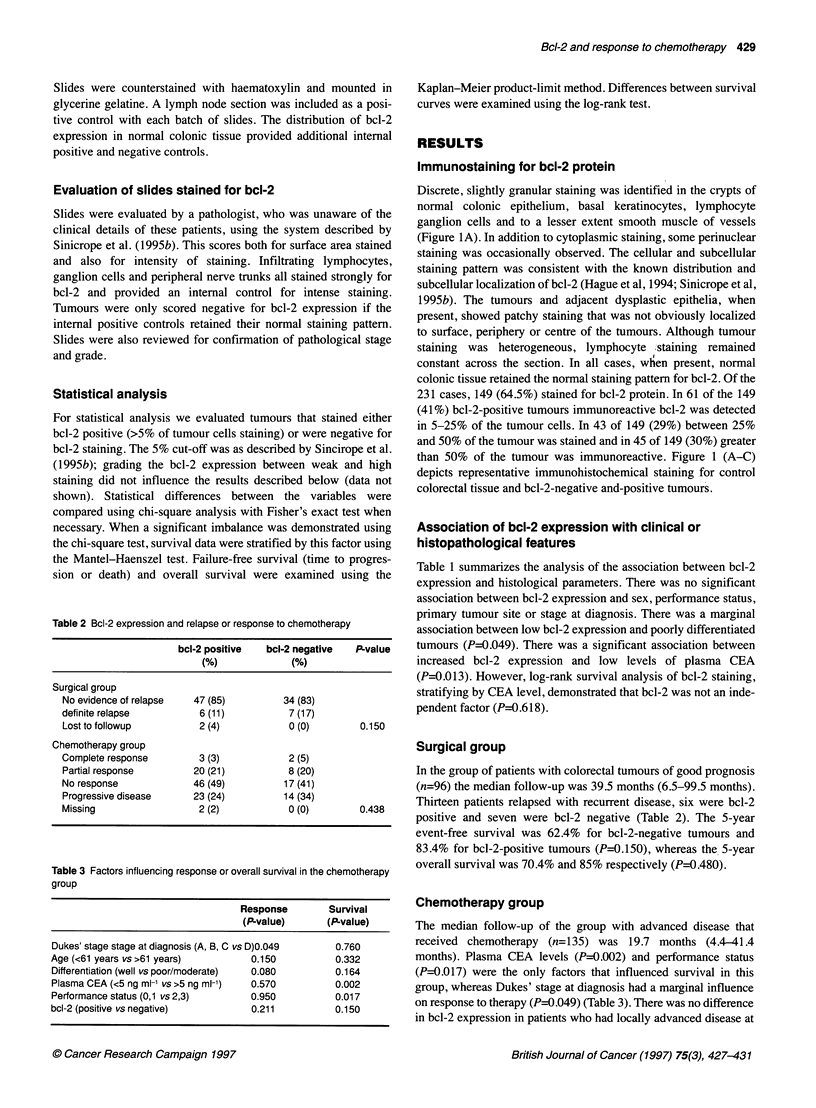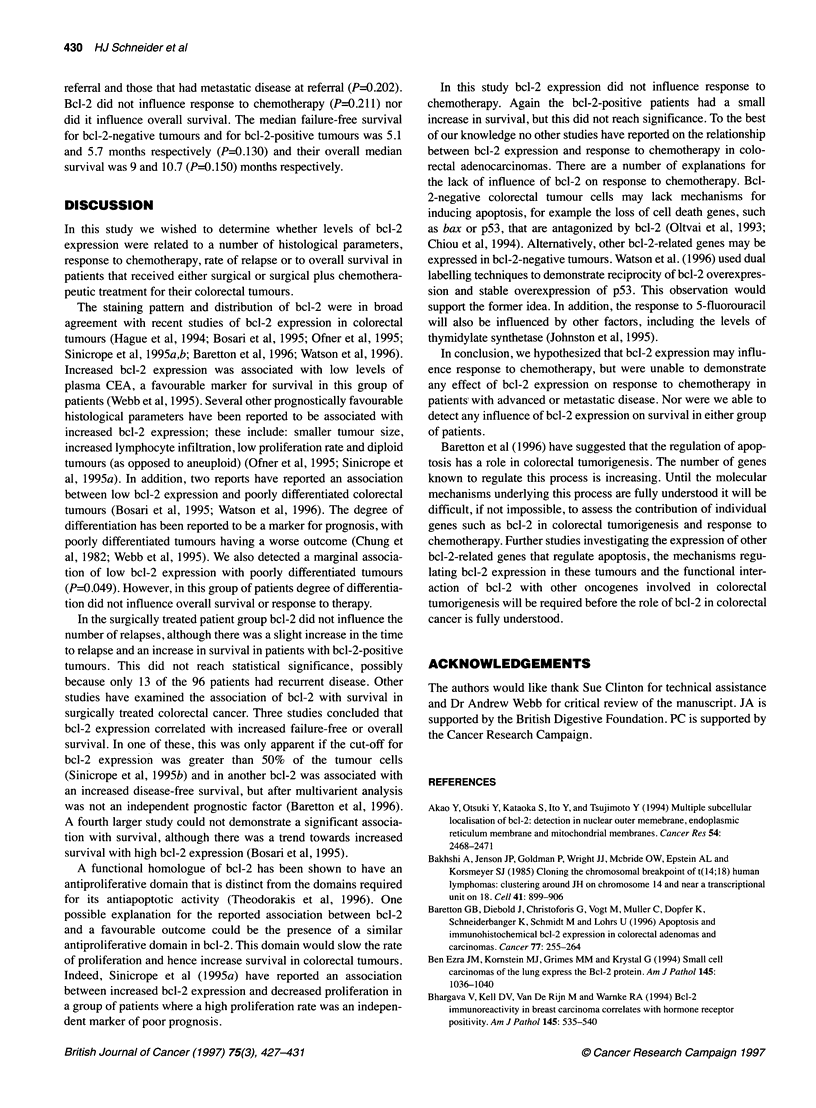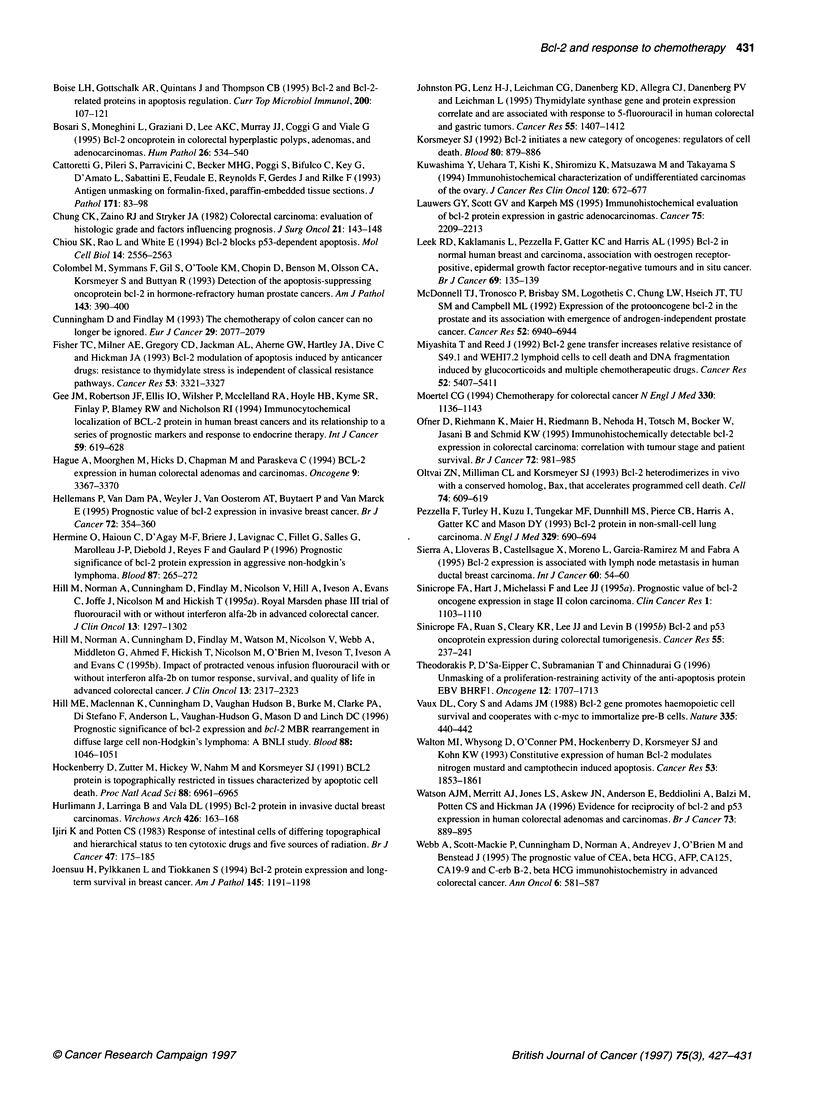# Bcl-2 expression and response to chemotherapy in colorectal adenocarcinomas.

**DOI:** 10.1038/bjc.1997.70

**Published:** 1997

**Authors:** H. J. Schneider, S. A. Sampson, D. Cunningham, A. R. Norman, H. J. Andreyev, J. V. Tilsed, P. A. Clarke

**Affiliations:** The Gastrointestinal Unit, Department of Medicine, Royal Marsden Hospital, Sutton, Surrey, UK.

## Abstract

**Images:**


British Journal of Cancer (1997) 75(3), 427–431
© 1997 Cancer Research Campaign

### Bcl-2 expression and response to chemotherapy in colorectal adenocarcinomas

**HJ Schneider[1*], SA Sampson[2], D Cunningham[1], AR Norman[3], HJN Andreyev[1,4], JVT Tilsed[1] and PA Clarke[4]**

[1]The Gastrointestinal Unit, Department of Medicine,[2]Department of Histopathology, [3]Department of Computing and Information, Royal Marsden Hospital and [4]Cancer Research Campaign Centre for Cancer Therapeutics, Institute of Cancer Research, Sutton, Surrey, UK

**Summary** In the last year, a number of studies have reported the expression of bcl-2 in colorectal adenocarcinomas. However, the influence of bcl-2 expression on response to chemotherapy and outcome in patients with advanced colorectal adenocarcinoma has not been reported. We analysed bcl-2 expression in 231 colorectal tumours from patients that were treated by surgery alone or with 5-fluorouracil-based chemotherapy. Of 231 tumours, 149 (64.5%) overexpressed bcl-2. Bcl-2 expression was associated with low plasma CEA levels ($P$=0.013) and inversely associated with poor differentiation ($P$=0.049). However, bcl-2 expression did not significantly influence failure-free or overall survival in surgically treated patients. In the group of patients receiving 5-fluorouracil-based chemotherapy bcl-2 expression did not influence response to chemotherapy; nor did it effect failure-free or overall survival.

**Key words:** bcl-2; chemotherapy; 5-fluorouracil; colorectal cancer

The *bcl-2* oncogene was originally identified through its association with the t(14;18) chromosomal translocation common in low-grade lymphomas (Bakhshi et al, 1985). The precise biochemical function of bcl-2 remains unknown, although sequence comparison, and in vitro and in vivo studies have shown that it belongs to a family of proteins that regulate programmed cell death (apoptosis) (Vaux et al, 1988; McDonnell et al, 1992; Boise et al, 1995). In vitro studies have also demonstrated that expression of bcl-2 results in the acquisition of resistance to a variety of agents that induce apoptosis (Miyashita and Reed, 1992; Fisher et al, 1993; Walton et al, 1993). Bcl-2 is expressed widely during embryogenesis and also in a number of normal adult tissues, including stem cells, peripheral neurones and some lymphoid cells (Hockenberry et al, 1991). The protein is mainly localized to mitochondrial and nuclear membranes and to the endoplasmic reticulum (Akao et al, 1994).

Bcl-2 expression has been detected in a number of tumours including breast (Bhargava et al, 1994; Joensuu et al, 1994; Hellemans et al, 1995; Sierra et al, 1995; Leek et al, 1995), colon (Hague et al, 1994; Bosari et al, 1995; Ofner et al, 1995; Sinicrope et al, 1995*a,b*; Baretton et al, 1996; Watson et al, 1996), gastric (Lauwers et al, 1995), lung (Pezzella et al, 1993; Ben Ezra et al, 1994), lymphoma (Hermine et al, 1996; Hill et al, 1996), ovary (Kuwashima et al, 1994) and prostate (McDonnell et al, 1992; Colombel et al, 1993). We and others have recently shown that bcl-2 expression in patients with diffuse large-cell lymphomas is associated with an increased rate of relapse and a significantly worse prognosis (Hermine et al, 1996; Hill et al, 1996). In contrast, there are some reports in breast tumours that suggest that tumours expressing bcl-2 have a better prognosis and are more responsive to endocrine therapy (Gee et al, 1994; Hellemans et al, 1995; Hurlimann et al, 1995).

A number of studies have examined the association of bcl-2 expression with a variety of histopathological and clinical parameters in colorectal adenocarcinoma (Bosari et al, 1995; Ofner et al, 1995;

*Received 19 July 1996*
*Revised 3 September 1996*
*Accepted 3 September 1996*

*Correspondence to:* D Cunningham, Department of Medicine, Royal Marsden Hospital Downs Rd, Sutton, Surrey SM2 5PT UK
*Present address: Department of Surgery, Queen Mary's Hospital, Sidcup, Kent, UK

**Table 1** Patient characteristics and bcl-2 expression

| | bcl-2 positive (%) | bcl-2 negative (%) | $P$-value |
|---|---|---|---|
| Sex | | | |
| M | 83 (58) | 47 (57) | |
| F | 66 (44) | 35 (43) | 0.813 |
| Performance status | | | |
| 0 | 19 (13) | 7 (9) | |
| 1 | 40 (27) | 20 (24) | |
| 2 | 30 (20) | 10 (12) | |
| 3 | 5 (3) | 4 (5) | |
| Unknown | 55 (37) | 41 (50) | 0.251 |
| Dukes' staging | | | |
| A | 50 (34) | 37 (45) | |
| B | 28 (19) | 8 (10) | |
| C | 31 (21) | 18 (22) | |
| D | 39 (26) | 19 (23) | |
| Missing | 1 (1) | 0 (0) | 0.181 |
| Differentiation | | | |
| Well/moderate | 139 (93) | 70 (85) | |
| Poor | 10 (7) | 12 (15) | 0.049 |
| Tumour site | | | |
| Right hemicolon | 26 (17) | 14 (17) | |
| Transverse colon | 9 (6) | 3 (4) | |
| Left hemicolon | 8 (5) | 6 (7) | |
| Sigmoid colon/rectum | 100 (67) | 54 (66) | |
| Unknown | 6 (4) | 5 (6) | 0.845 |
| CEA | | | |
| >5 ng ml$^{-1}$ | 67 (45) | 34 (41) | |
| <5 ng ml$^{-1}$ | 19 (13) | 2 (2) | |
| Unknown | 63 (42) | 46 (56) | 0.013 |

**Figure 1** Bcl-2 staining of normal colorectal tissue and colorectal tumours. **(A)** Normal colorectal tissue showing staining of the crypts and adjacent lymphocytes (original magnification × 200). **(B)** Bcl-2-negative colorectal carcinoma with staining of internal positive control lymphocytes (original magnification × 200). **(C)** Bcl-2-positive tubulovillous colorectal carcinoma (original magnification × 200)

Sinicrope et al, 1995*a,b*; Baretton et al, 1996). Three studies concluded that bcl-2 expression was associated with increased survival (Ofner et al, 1995; Sinicrope et al, 1995; Baretton et al, 1996), an observation similar to that seen in breast tumours. However, a larger study could not detect any association between survival and bcl-2 expression (Bosari et al, 1995). None of these studies have examined the influence of bcl-2 expression on response to chemotherapy. In three of these studies the patients were treated by surgery alone (Ofner et al, 1995; Sinicrope et al, 1995; Baretton et al, 1996) and in the fourth study there was no prior chemotherapy (Bosari et al, 1995), whereas treatment following surgery was not reported.

A drug often used in the adjuvant and palliative treatment of colorectal tumours is 5-fluorouracil (Cunningham and Findlay, 1993; Moertel, 1994). This agent inhibits nucleic acid biosynthesis by a number of different mechanisms and induces apoptosis both in vitro and in vivo (Ijiri and Potten, 1983; Fisher et al, 1993). In vitro studies have demonstrated that bcl-2 can inhibit apoptosis induced by 5-fluorouracil (Fisher et al, 1993). Therefore, we were interested in the influence of bcl-2 on response to 5-fluorouracil-based chemotherapeutic regimens in patients with colorectal tumours. The first aim of this study was to investigate the association between bcl-2 expression and outcome in colorectal cancer. The second aim was to examine whether response and outcome following chemotherapy were altered in those patients who expressed bcl-2.

### MATERIALS AND METHODS

### Patients

Slides were cut from paraffin-embedded tissue resected from patients with colorectal tumours at time of diagnosis. We were able to identify archive material from 231 patients, whose details are summarized in Table 1. Patients had either been treated by surgery, with a combination of surgery and chemotherapy or with chemotherapy alone. A total of 96 patients had Dukes' A and B colorectal tumours at diagnosis that were considered not to require adjuvant chemotherapy. They were treated by surgical resection alone. A total of 135 patients had advanced or metastatic disease. They had received no prior chemotherapy or radiotherapy and were enrolled in two randomized clinical trials at the Royal Marsden Hospital (Hill et al, 1995*a,b*). These patients received either bolus or protracted venous infusional 5-fluorouracil ± interferon. The criteria for inclusion in these studies, for response and for follow-up are detailed elsewhere (Hill et al, 1995*a,b*).

### Bcl-2 immunohistochemistry

Sections (3 μm) were deparaffinized in Histoclear and rehydrated in 100% ethanol. Endogenous peroxidase activity was quenched with 20% acetic acid. Slides were immersed in citrate buffer solution pH 6.0 and were microwaved for 10 min in a 850-W microwave (Cattoretti et al, 1993). The samples were cooled for 20 min and rinsed in tris-buffered saline. Slides were blocked with 5% horse serum in phosphate-buffered saline (PBS) for 20 min and incubated in a humidified chamber for 90 min at room temperature with a bcl-2 antibody (2.5 μg $ml^{-1}$ of monoclonal antibody no. 100, Oncogene Science) diluted in PBS/0.5% bovine serum albumin. Bcl-2–antibody complexes were detected using an APAAP system (Vector Laboratories) following the manufacturer's instructions.

© Cancer Research Campaign 1997

Slides were counterstained with haematoxylin and mounted in glycerine gelatine. A lymph node section was included as a positive control with each batch of slides. The distribution of bcl-2 expression in normal colonic tissue provided additional internal positive and negative controls.

### Evaluation of slides stained for bcl-2

Slides were evaluated by a pathologist, who was unaware of the clinical details of these patients, using the system described by Sinicrope et al. (1995*b*). This scores both for surface area stained and also for intensity of staining. Infiltrating lymphocytes, ganglion cells and peripheral nerve trunks all stained strongly for bcl-2 and provided an internal control for intense staining. Tumours were only scored negative for bcl-2 expression if the internal positive controls retained their normal staining pattern. Slides were also reviewed for confirmation of pathological stage and grade.

### Statistical analysis

For statistical analysis we evaluated tumours that stained either bcl-2 positive (>5% of tumour cells staining) or were negative for bcl-2 staining. The 5% cut-off was as described by Sincirope et al. (1995*b*); grading the bcl-2 expression between weak and high staining did not influence the results described below (data not shown). Statistical differences between the variables were compared using chi-square analysis with Fisher's exact test when necessary. When a significant imbalance was demonstrated using the chi-square test, survival data were stratified by this factor using the Mantel–Haenszel test. Failure-free survival (time to progression or death) and overall survival were examined using the Kaplan–Meier product-limit method. Differences between survival curves were examined using the log-rank test.

**Table 2** Bcl-2 expression and relapse or response to chemotherapy

| | bcl-2 positive (%) | bcl-2 negative (%) | *P*-value |
|---|---|---|---|
| Surgical group | | | |
| No evidence of relapse | 47 (85) | 34 (83) | |
| definite relapse | 6 (11) | 7 (17) | |
| Lost to followup | 2 (4) | 0 (0) | 0.150 |
| Chemotherapy group | | | |
| Complete response | 3 (3) | 2 (5) | |
| Partial response | 20 (21) | 8 (20) | |
| No response | 46 (49) | 17 (41) | |
| Progressive disease | 23 (24) | 14 (34) | |
| Missing | 2 (2) | 0 (0) | 0.438 |

**Table 3** Factors influencing response or overall survival in the chemotherapy group

| | Response (*P*-value) | Survival (*P*-value) |
|---|---|---|
| Dukes' stage stage at diagnosis (A, B, C *vs* D) | 0.049 | 0.760 |
| Age (<61 years *vs* >61 years) | 0.150 | 0.332 |
| Differentiation (well *vs* poor/moderate) | 0.080 | 0.164 |
| Plasma CEA (<5 ng $ml^{-1}$ *vs* >5 ng $ml^{-1}$) | 0.570 | 0.002 |
| Performance status (0,1 *vs* 2,3) | 0.950 | 0.017 |
| bcl-2 (positive *vs* negative) | 0.211 | 0.150 |

### RESULTS

### Immunostaining for bcl-2 protein

Discrete, slightly granular staining was identified in the crypts of normal colonic epithelium, basal keratinocytes, lymphocyte ganglion cells and to a lesser extent smooth muscle of vessels (Figure 1A). In addition to cytoplasmic staining, some perinuclear staining was occasionally observed. The cellular and subcellular staining pattern was consistent with the known distribution and subcellular localization of bcl-2 (Hague et al, 1994; Sinicrope et al, 1995*b*). The tumours and adjacent dysplastic epithelia, when present, showed patchy staining that was not obviously localized to surface, periphery or centre of the tumours. Although tumour staining was heterogeneous, lymphocyte staining remained constant across the section. In all cases, when present, normal colonic tissue retained the normal staining pattern for bcl-2. Of the 231 cases, 149 (64.5%) stained for bcl-2 protein. In 61 of the 149 (41%) bcl-2-positive tumours immunoreactive bcl-2 was detected in 5–25% of the tumour cells. In 43 of 149 (29%) between 25% and 50% of the tumour was stained and in 45 of 149 (30%) greater than 50% of the tumour was immunoreactive. Figure 1 (A–C) depicts representative immunohistochemical staining for control colorectal tissue and bcl-2-negative and-positive tumours.

### Association of bcl-2 expression with clinical or histopathological features

Table 1 summarizes the analysis of the association between bcl-2 expression and histological parameters. There was no significant association between bcl-2 expression and sex, performance status, primary tumour site or stage at diagnosis. There was a marginal association between low bcl-2 expression and poorly differentiated tumours ($P$=0.049). There was a significant association between increased bcl-2 expression and low levels of plasma CEA ($P$=0.013). However, log-rank survival analysis of bcl-2 staining, stratifying by CEA level, demonstrated that bcl-2 was not an independent factor ($P$=0.618).

### Surgical group

In the group of patients with colorectal tumours of good prognosis ($n$=96) the median follow-up was 39.5 months (6.5–99.5 months). Thirteen patients relapsed with recurrent disease, six were bcl-2 positive and seven were bcl-2 negative (Table 2). The 5-year event-free survival was 62.4% for bcl-2-negative tumours and 83.4% for bcl-2-positive tumours ($P$=0.150), whereas the 5-year overall survival was 70.4% and 85% respectively ($P$=0.480).

### Chemotherapy group

The median follow-up of the group with advanced disease that received chemotherapy ($n$=135) was 19.7 months (4.4–41.4 months). Plasma CEA levels ($P$=0.002) and performance status ($P$=0.017) were the only factors that influenced survival in this group, whereas Dukes' stage at diagnosis had a marginal influence on response to therapy ($P$=0.049) (Table 3). There was no difference in bcl-2 expression in patients who had locally advanced disease at

© Cancer Research Campaign 1997

referral and those that had metastatic disease at referral ($P$=0.202). Bcl-2 did not influence response to chemotherapy ($P$=0.211) nor did it influence overall survival. The median failure-free survival for bcl-2-negative tumours and for bcl-2-positive tumours was 5.1 and 5.7 months respectively ($P$=0.130) and their overall median survival was 9 and 10.7 ($P$=0.150) months respectively.

### DISCUSSION

In this study we wished to determine whether levels of bcl-2 expression were related to a number of histological parameters, response to chemotherapy, rate of relapse or to overall survival in patients that received either surgical or surgical plus chemotherapeutic treatment for their colorectal tumours.

The staining pattern and distribution of bcl-2 were in broad agreement with recent studies of bcl-2 expression in colorectal tumours (Hague et al, 1994; Bosari et al, 1995; Ofner et al, 1995; Sinicrope et al, 1995*a,b*; Baretton et al, 1996; Watson et al, 1996). Increased bcl-2 expression was associated with low levels of plasma CEA, a favourable marker for survival in this group of patients (Webb et al, 1995). Several other prognostically favourable histological parameters have been reported to be associated with increased bcl-2 expression; these include: smaller tumour size, increased lymphocyte infiltration, low proliferation rate and diploid tumours (as opposed to aneuploid) (Ofner et al, 1995; Sinicrope et al, 1995*a*). In addition, two reports have reported an association between low bcl-2 expression and poorly differentiated colorectal tumours (Bosari et al, 1995; Watson et al, 1996). The degree of differentiation has been reported to be a marker for prognosis, with poorly differentiated tumours having a worse outcome (Chung et al, 1982; Webb et al, 1995). We also detected a marginal association of low bcl-2 expression with poorly differentiated tumours ($P$=0.049). However, in this group of patients degree of differentiation did not influence overall survival or response to therapy.

In the surgically treated patient group bcl-2 did not influence the number of relapses, although there was a slight increase in the time to relapse and an increase in survival in patients with bcl-2-positive tumours. This did not reach statistical significance, possibly because only 13 of the 96 patients had recurrent disease. Other studies have examined the association of bcl-2 with survival in surgically treated colorectal cancer. Three studies concluded that bcl-2 expression correlated with increased failure-free or overall survival. In one of these, this was only apparent if the cut-off for bcl-2 expression was greater than 50% of the tumour cells (Sinicrope et al, 1995*b*) and in another bcl-2 was associated with an increased disease-free survival, but after multivarient analysis was not an independent prognostic factor (Baretton et al, 1996). A fourth larger study could not demonstrate a significant association with survival, although there was a trend towards increased survival with high bcl-2 expression (Bosari et al, 1995).

A functional homologue of bcl-2 has been shown to have an antiproliferative domain that is distinct from the domains required for its antiapoptotic activity (Theodorakis et al, 1996). One possible explanation for the reported association between bcl-2 and a favourable outcome could be the presence of a similar antiproliferative domain in bcl-2. This domain would slow the rate of proliferation and hence increase survival in colorectal tumours. Indeed, Sinicrope et al (1995*a*) have reported an association between increased bcl-2 expression and decreased proliferation in a group of patients where a high proliferation rate was an independent marker of poor prognosis.

In this study bcl-2 expression did not influence response to chemotherapy. Again the bcl-2-positive patients had a small increase in survival, but this did not reach significance. To the best of our knowledge no other studies have reported on the relationship between bcl-2 expression and response to chemotherapy in colorectal adenocarcinomas. There are a number of explanations for the lack of influence of bcl-2 on response to chemotherapy. Bcl-2-negative colorectal tumour cells may lack mechanisms for inducing apoptosis, for example the loss of cell death genes, such as *bax* or p53, that are antagonized by bcl-2 (Oltvai et al, 1993; Chiou et al, 1994). Alternatively, other bcl-2-related genes may be expressed in bcl-2-negative tumours. Watson et al. (1996) used dual labelling techniques to demonstrate reciprocity of bcl-2 overexpression and stable overexpression of p53. This observation would support the former idea. In addition, the response to 5-fluorouracil will also be influenced by other factors, including the levels of thymidylate synthetase (Johnston et al, 1995).

In conclusion, we hypothesized that bcl-2 expression may influence response to chemotherapy, but were unable to demonstrate any effect of bcl-2 expression on response to chemotherapy in patients with advanced or metastatic disease. Nor were we able to detect any influence of bcl-2 expression on survival in either group of patients.

Baretton et al (1996) have suggested that the regulation of apoptosis has a role in colorectal tumorigenesis. The number of genes known to regulate this process is increasing. Until the molecular mechanisms underlying this process are fully understood it will be difficult, if not impossible, to assess the contribution of individual genes such as bcl-2 in colorectal tumorigenesis and response to chemotherapy. Further studies investigating the expression of other bcl-2-related genes that regulate apoptosis, the mechanisms regulating bcl-2 expression in these tumours and the functional interaction of bcl-2 with other oncogenes involved in colorectal tumorigenesis will be required before the role of bcl-2 in colorectal cancer is fully understood.

### ACKNOWLEDGEMENTS

The authors would like thank Sue Clinton for technical assistance and Dr Andrew Webb for critical review of the manuscript. JA is supported by the British Digestive Foundation. PC is supported by the Cancer Research Campaign.

### REFERENCES

Akao Y, Otsuki Y, Kataoka S, Ito Y, and Tsujimoto Y (1994) Multiple subcellular localisation of bcl-2: detection in nuclear outer memebrane, endoplasmic reticulum membrane and mitochondrial membranes. *Cancer Res* **54**: 2468–2471

Bakhshi A, Jenson JP, Goldman P, Wright JJ, Mcbride OW, Epstein AL and Korsmeyer SJ (1985) Cloning the chromosomal breakpoint of t(14;18) human lymphomas: clustering around JH on chromosome 14 and near a transcriptional unit on 18. *Cell* **41**: 899–906

Baretton GB, Diebold J, Christoforis G, Vogt M, Muller C, Dopfer K, Schneiderbanger K, Schmidt M and Lohrs U (1996) Apoptosis and immunohistochemical bcl-2 expression in colorectal adenomas and carcinomas. *Cancer* **77**: 255–264

Ben Ezra JM, Kornstein MJ, Grimes MM and Krystal G (1994) Small cell carcinomas of the lung express the Bcl-2 protein. *Am J Pathol* **145**: 1036–1040

Bhargava V, Kell DV, Van De Rijn M and Warnke RA (1994) Bcl-2 immunoreactivity in breast carcinoma correlates with hormone receptor positivity. *Am J Pathol* **145**: 535–540

© Cancer Research Campaign 1997

Boise LH, Gottschalk AR, Quintans J and Thompson CB (1995) Bcl-2 and Bcl-2-related proteins in apoptosis regulation. *Curr Top Microbiol Immunol*, **200**: 107–121

Bosari S, Moneghini L, Graziani D, Lee AKC, Murray JJ, Coggi G and Viale G (1995) Bcl-2 oncoprotein in colorectal hyperplastic polyps, adenomas, and adenocarcinomas. *Hum Pathol* **26**: 534–540

Cattoretti G, Pileri S, Parravicini C, Becker MHG, Poggi S, Bifulco C, Key G, D'Amato L, Sabattini E, Feudale E, Reynolds F, Gerdes J and Rilke F (1993) Antigen unmasking on formalin-fixed, paraffin-embedded tissue sections. *J Pathol* **171**: 83–98

Chung CK, Zaino RJ and Stryker JA (1982) Colorectal carcinoma: evaluation of histologic grade and factors influencing prognosis. *J Surg Oncol* **21**: 143–148

Chiou SK, Rao L and White E (1994) Bcl-2 blocks p53-dependent apoptosis. *Mol Cell Biol* **14**: 2556–2563

Colombel M, Symmans F, Gil S, O'Toole KM, Chopin D, Benson M, Olsson CA, Korsmeyer S and Buttyan R (1993) Detection of the apoptosis-suppressing oncoprotein bcl-2 in hormone-refractory human prostate cancers. *Am J Pathol* **143**: 390–400

Cunningham D and Findlay M (1993) The chemotherapy of colon cancer can no longer be ignored. *Eur J Cancer* **29**: 2077–2079

Fisher TC, Milner AE, Gregory CD, Jackman AL, Aherne GW, Hartley JA, Dive C and Hickman JA (1993) Bcl-2 modulation of apoptosis induced by anticancer drugs: resistance to thymidylate stress is independent of classical resistance pathways. *Cancer Res* **53**: 3321–3327

Gee JM, Robertson JF, Ellis IO, Wilsher P, Mcclelland RA, Hoyle HB, Kyme SR, Finlay P, Blamey RW and Nicholson RI (1994) Immunocytochemical localization of BCL-2 protein in human breast cancers and its relationship to a series of prognostic markers and response to endocrine therapy. *Int J Cancer* **59**: 619–628

Hague A, Moorghen M, Hicks D, Chapman M and Paraskeva C (1994) BCL-2 expression in human colorectal adenomas and carcinomas. *Oncogene* **9**: 3367–3370

Hellemans P, Van Dam PA, Weyler J, Van Oosterom AT, Buytaert P and Van Marck E (1995) Prognostic value of bcl-2 expression in invasive breast cancer. *Br J Cancer* **72**: 354–360

Hermine O, Haioun C, D'Agay M-F, Briere J, Lavignac C, Fillet G, Salles G, Marolleau J-P, Diebold J, Reyes F and Gaulard P (1996) Prognostic significance of bcl-2 protein expression in aggressive non-hodgkin's lymphoma. *Blood* **87**: 265–272

Hill M, Norman A, Cunningham D, Findlay M, Nicolson V, Hill A, Iveson A, Evans C, Joffe J, Nicolson M and Hickish T (1995*a*). Royal Marsden phase III trial of fluorouracil with or without interferon alfa-2b in advanced colorectal cancer. *J Clin Oncol* **13**: 1297–1302

Hill M, Norman A, Cunningham D, Findlay M, Watson M, Nicolson V, Webb A, Middleton G, Ahmed F, Hickish T, Nicolson M, O'Brien M, Iveson T, Iveson A and Evans C (1995b). Impact of protracted venous infusion fluorouracil with or without interferon alfa-2b on tumor response, survival, and quality of life in advanced colorectal cancer. *J Clin Oncol* **13**: 2317–2323

Hill ME, Maclennan K, Cunningham D, Vaughan Hudson B, Burke M, Clarke PA, Di Stefano F, Anderson L, Vaughan-Hudson G, Mason D and Linch DC (1996) Prognostic significance of bcl-2 expression and *bcl-2* MBR rearrangement in diffuse large cell non-Hodgkin's lymphoma: A BNLI study. *Blood* **88:** 1046–1051

Hockenberry D, Zutter M, Hickey W, Nahm M and Korsmeyer SJ (1991) BCL2 protein is topographically restricted in tissues characterized by apoptotic cell death. *Proc Natl Acad Sci* **88**: 6961–6965

Hurlimann J, Larringa B and Vala DL (1995) Bcl-2 protein in invasive ductal breast carcinomas. *Virchows Arch* **426**: 163–168

Ijiri K and Potten CS (1983) Response of intestinal cells of differing topographical and hierarchical status to ten cytotoxic drugs and five sources of radiation. *Br J Cancer* **47**: 175–185

Joensuu H, Pylkkanen L and Tiokkanen S (1994) Bcl-2 protein expression and long-term survival in breast cancer. *Am J Pathol* **145**: 1191–1198

Johnston PG, Lenz H-J, Leichman CG, Danenberg KD, Allegra CJ, Danenberg PV and Leichman L (1995) Thymidylate synthase gene and protein expression correlate and are associated with response to 5-fluorouracil in human colorectal and gastric tumors. *Cancer Res* **55**: 1407–1412

Korsmeyer SJ (1992) Bcl-2 initiates a new category of oncogenes: regulators of cell death. *Blood* **80**: 879–886

Kuwashima Y, Uehara T, Kishi K, Shiromizu K, Matsuzawa M and Takayama S (1994) Immunohistochemical characterization of undifferentiated carcinomas of the ovary. *J Cancer Res Clin Oncol* **120**: 672–677

Lauwers GY, Scott GV and Karpeh MS (1995) Immunohistochemical evaluation of bcl-2 protein expression in gastric adenocarcinomas. *Cancer* **75**: 2209–2213

Leek RD, Kaklamanis L, Pezzella F, Gatter KC and Harris AL (1995) Bcl-2 in normal human breast and carcinoma, association with oestrogen receptor-positive, epidermal growth factor receptor-negative tumours and in situ cancer. *Br J Cancer* **69**: 135–139

McDonnell TJ, Tronosco P, Brisbay SM, Logothetis C, Chung LW, Hseich JT, TU SM and Campbell ML (1992) Expression of the protooncogene bcl-2 in the prostate and its association with emergence of androgen-independent prostate cancer. *Cancer Res* **52**: 6940–6944

Miyashita T and Reed J (1992) Bcl-2 gene transfer increases relative resistance of S49.1 and WEHI7.2 lymphoid cells to cell death and DNA fragmentation induced by glucocorticoids and multiple chemotherapeutic drugs. *Cancer Res* **52**: 5407–5411

Moertel CG (1994) Chemotherapy for colorectal cancer *N Engl J Med* **330**: 1136–1143

Ofner D, Riehmann K, Maier H, Riedmann B, Nehoda H, Totsch M, Bocker W, Jasani B and Schmid KW (1995) Immunohistochemically detectable bcl-2 expression in colorectal carcinoma: correlation with tumour stage and patient survival. *Br J Cancer* **72**: 981–985

Oltvai ZN, Milliman CL and Korsmeyer SJ (1993) Bcl-2 heterodimerizes in vivo with a conserved homolog, Bax, that accelerates programmed cell death. *Cell* **74**: 609–619

Pezzella F, Turley H, Kuzu I, Tungekar MF, Dunnhill MS, Pierce CB, Harris A, Gatter KC and Mason DY (1993) Bcl-2 protein in non-small-cell lung carcinoma. *N Engl J Med* **329**: 690–694

Sierra A, Lloveras B, Castellsague X, Moreno L, Garcia-Ramirez M and Fabra A (1995) Bcl-2 expression is associated with lymph node metastasis in human ductal breast carcinoma. *Int J Cancer* **60**: 54–60

Sinicrope FA, Hart J, Michelassi F and Lee JJ (1995*a*). Prognostic value of bcl-2 oncogene expression in stage II colon carcinoma. *Clin Cancer Res* **1**: 1103–1110

Sinicrope FA, Ruan S, Cleary KR, Lee JJ and Levin B (1995*b*) Bcl-2 and p53 oncoprotein expression during colorectal tumorigenesis. *Cancer Res* **55**: 237–241

Theodorakis P, D'Sa-Eipper C, Subramanian T and Chinnadurai G (1996) Unmasking of a proliferation-restraining activity of the anti-apoptosis protein EBV BHRF1. *Oncogene* **12**: 1707–1713

Vaux DL, Cory S and Adams JM (1988) Bcl-2 gene promotes haemopoietic cell survival and cooperates with c-myc to immortalize pre-B cells. *Nature* **335**: 440–442

Walton MI, Whysong D, O'Conner PM, Hockenberry D, Korsmeyer SJ and Kohn KW (1993) Constitutive expression of human Bcl-2 modulates nitrogen mustard and camptothecin induced apoptosis. *Cancer Res* **53**: 1853–1861

Watson AJM, Merritt AJ, Jones LS, Askew JN, Anderson E, Beddiolini A, Balzi M, Potten CS and Hickman JA (1996) Evidence for reciprocity of bcl-2 and p53 expression in human colorectal adenomas and carcinomas. *Br J Cancer* **73**: 889–895

Webb A, Scott-Mackie P, Cunningham D, Norman A, Andreyev J, O'Brien M and Benstead J (1995) The prognostic value of CEA, beta HCG, AFP, CA125, CA19-9 and C-erb B-2, beta HCG immunohistochemistry in advanced colorectal cancer. *Ann Oncol* **6**: 581–587

© Cancer Research Campaign 1997